# Acceptance of a malaria vaccine by caregivers of sick children in Kenya

**DOI:** 10.1186/1475-2875-13-172

**Published:** 2014-05-05

**Authors:** David I Ojakaa, Jordan D Jarvis, Mary I Matilu, Sylla Thiam

**Affiliations:** 1 AMREF Kenya, PO Box 30125, 00100 Nairobi, Kenya; 2 AMREF Canada, 489 College Street West, Suite 403, Toronto, ON M6G 1A5, Canada; 3 Kenya Medical Research Institute, PO Box 54840, 00200 Nairobi, Kenya; 4 AMREF Headquarters, PO Box 27691, 00506 Nairobi, Kenya

**Keywords:** Malaria vaccine, Acceptance, Caregivers, Kenya

## Abstract

**Background:**

Several malaria vaccines are currently in clinical trials and are expected to provide an improved strategy for malaria control. Prior to introduction of a new vaccine, policymakers must consider the socio cultural environment of the region to ensure widespread community approval. This study investigated the acceptance of a malaria vaccine by child caregivers and analysed factors that influence these.

**Methods:**

Interviews from a standard questionnaire were conducted with 2,003 caregivers at 695 randomly selected health facilities across Kenya during the Kenya Service Provision Assessment Survey 2010. Multinomial regression of quantitative data was conducted using STATA to analyse determinants of caregivers accepting malaria vaccination of their child.

**Results:**

Mothers represented 90% of caregivers interviewed who brought their child to the health facility, and 77% of caregivers were 20-34 years old. Overall, 88% of respondents indicated that they would accept a malaria vaccine, both for a child in their community and their own child. Approval for a vaccine was highest in malaria-endemic Nyanza Province at 98.9%, and lowest in the seasonal transmission area of North Eastern Province at 23%. Although 94% of respondents who had attended at least some school reported they would accept the vaccine for a child, only 56% of those who had never attended school would do so. The likelihood of accepting one’s own child to be immunized was correlated with province, satisfaction with health care services in the facility attended, age of the caregiver, and level of education.

**Conclusions:**

Results from this study indicate a need for targeted messages and education on a malaria vaccine, particularly for residents of regions where acceptance is low, older caregivers, and those with low literacy and school-attendance levels. This study provides critical evidence to inform policy for a new malaria vaccine that will support its timely and comprehensive uptake in Kenya.

## Background

While childhood mortality is decreasing in Africa as a whole, malaria-specific mortality persists as a heavy public health burden, particularly in sub-Saharan Africa. Multiple malaria interventions, including vector control measures, intermittent preventative treatment, and anti-malarial drugs have long been in use to prevent or treat malaria. Even though notable progress has been made, more work is needed and new challenges are emerging. Drug resistance as well as insecticide-resistant mosquitoes, are growing threats in many areas. This is illustrated by the re-emergence of the disease in areas that had previously seen reductions in the malaria burden and been malaria-free [1,2]. This represents a major threat for malaria control and elimination, illuminating the need to develop new tools in order to re-enforce the package of existing interventions.

Development of a malaria vaccine is envisaged to provide a cost-effective reduction of the disease. Current progress toward developing a malaria vaccine has accelerated in the last decade with increased funding and research driving the discovery of new antigens and vaccine technology, and many more malaria vaccine candidates being moved through the development pipeline. The future of malaria vaccine development is promising particularly with RTS,S (also known as RTS, S/AS) which is the most clinically advanced malaria vaccine candidate in the world. It is a recombinant protein that fuses a part of the *Plasmodium falciparum* circumsporozoite protein with the hepatitis B virus surface antigen, combined with an adjuvant. RTS,S vaccine induces the production of antibodies and T cells that are believed to diminish the malaria parasite’s ability to infect, develop, and survive in the human liver. The vaccine is undergoing phase III clinical trials in seven African countries (Burkina Faso, Gabon, Ghana, Kenya, Malawi, Mozambique, and Tanzania), with 15,460 infants and young children participating. Results in African children showed that it protects 31% of young infants and 56% of older babies and toddlers from *P. falciparum*, the most life-threatening malaria parasite [3,4], but efficacy declined to 29.9% and 16.8% against first and all episodes of *P. falciparum* clinical malaria respectively, among children vaccinated at 5 to 17 months of age during 4 years of follow-up at one Kenyan site [5]. The multicentre RTS,S assessment is expected to run until late 2014, and is being conducted in three regions in Kenya, in addition to nine sites in the other African countries. Furthermore, a recent study reported that another vaccine containing live sporozoites of *P. falciparum* demonstrated potential for high-level protection against malaria in Phase I clinical trials [6].

If the required public health information comprising safety and efficacy data, benefits and risks are deemed satisfactory, a policy recommendation for this vaccine is possible in 2015. Henceforth, this would pave the way for decisions within malaria-endemic countries to implement and scale-up the vaccine through their national immunization programmes. A Decision-Making Framework has been developed by the World Health Organization (WHO) and the PATH Malaria Vaccine Initiative (MVI) [7] to facilitate timely and informed decisions for malaria vaccine introduction into health systems in Africa. Once approved, it is expected that the vaccine will be distributed within endemic regions of sub-Saharan Africa for malaria prevention, and be used alongside existing interventions.

However, the introduction of all new interventions, and particularly vaccines, requires an understanding of the local socio-cultural environment. This has been highlighted by challenges in regions such as northern Nigeria, where polio vaccination programmes failed to be accepted by the community due to misconceptions held and perpetuated by religious leaders [8]. Various reasons, primarily associated with the vaccination programme itself, have been found to influence the rejection of vaccines by communities in which they have been introduced. Factors that have contributed to vaccine refusal in sub-Saharan Africa include: limited parental and community knowledge of immunization and/or lack of access to information on childhood immunization, insufficient information about target diseases, negative vaccine attitudes in the community, false perceptions on disease susceptibility and severity, poor experiences with health care providers, and general concerns about vaccination safety [9,10]. To improve vaccine acceptance and childhood immunization coverage in sub-Saharan Africa, public health programmes should be designed to address specific barriers faced by diverse people and communities. Assessing community perceptions of malaria vaccine is a key element of the Decision-Making Framework and would help guide public discussions and answer critical questions for strengthened decision-making on the introduction of a malaria vaccine. Under the leadership of MVI, qualitative studies have been conducted on community expectations and acceptability of the malaria vaccine in malaria-endemic regions in Kenya, Mozambique and Ghana [11-13]. In all three countries, there was widespread support for a potential malaria vaccine, provided it was deemed efficacious and became widely accessible.

It is estimated that about 24 million Kenyans are at risk of malaria [14]. A study conducted in two malaria endemic regions of Kenya, Busia and the South coast, found that communities supported local childhood immunization programmes, although understanding of vaccines and what they do was limited. These limitations in understanding were most pronounced among younger and older people, particularly men. The current practice to promote health education on child welfare and immunization is to target women. Findings of the aforementioned study demonstrate that communications strategies should be developed to target men and women equally and in gender-appropriate ways [13]. Furthermore, this should involve influential community members and provide needed information and reassurances about immunization. Efforts should also be made to address concerns about the quality of immunization services and include improving health workers’ interpersonal communication skills [15].

The aforementioned studies were focused on exploring socio cultural and health communication issues among individuals who are responsible for, or influence decisions about vaccine use. Furthermore, they were carried out in resource-limited settings with small, carefully selected qualitative samples. Additional nationwide quantitative data are needed with a focus on factors influencing the attitudes of child caregivers. Furthermore, an understanding of community perceptions and acceptability of a malaria vaccine would be particularly informative in countries where clinical trials for the vaccine are underway.

This study examined caregivers’ acceptability of a malaria vaccine by using selected determinants to plan for the introduction of a malaria vaccine.

## Methods

### Setting

Kenya has four malaria epidemiological zones, with diversity in risk determined largely by altitude, rainfall patterns and temperature. The zones are:

• **Endemic**

Areas of stable malaria have altitudes ranging from 0 to 1,300 m around Lake Victoria in western Kenya and in the coastal regions. Rainfall, temperature and humidity are the determinants of the perennial transmission of malaria. The vector life cycle is usually short and survival rates are high because of the suitable climatic conditions. Transmission is intense throughout the year, with annual entomological inoculation rates of 30–100 [16]. Western, Nyanza and Coast Provinces fall in these malaria-endemic zones.

• **Seasonal transmission**

Arid and semi-arid areas of northern and south-eastern parts of the country experience short periods of intense malaria transmission during the rainfall seasons. Temperatures are usually high and water pools created during the rainy season provide breeding sites for the malaria vectors. Extreme climatic conditions, such as the *El Niño* southern oscillation, lead to flooding in these areas, resulting in epidemic outbreaks with high morbidity rates owing to the low immune status of the population [16]. Eastern and North Eastern Provinces and parts of Central Province fall in this seasonal transmission zone.

• **Epidemic-prone areas of the Western highlands of Kenya**

Malaria transmission in the Western highlands of Kenya is seasonal, with considerable year-to-year variation. Epidemics are experienced when climatic conditions favour sustainability of minimum temperatures around 18°C. This increase in minimum temperatures during the long rains favours and sustains vector breeding, resulting in increased intensity of malaria transmission. The whole population is vulnerable and case fatality rates during an epidemic can be up to ten times greater than those experienced in regions where malaria occurs regularly [16]. Rift Valley Province and some parts of Nyanza Province fall in this zone.

• **Low risk malaria areas**

This zone covers the central highlands of Kenya, including Nairobi. The temperatures are usually too low to allow completion of the development cycle of the malaria parasite in the mosquito vector. However, the increasing temperatures and changes in the hydrological cycle associated with climate change are likely to increase the areas suitable for malaria vector breeding with the introduction of malaria transmission in areas where it had not existed before [17]. *Plasmodium falciparum* is the most prevalent parasite species and counts for 96% of malaria cases [16]. Nairobi Province and some parts of Central Province are considered malaria low risk zones.

### Study design and sample

Questions on the perceptions of caregivers of sick children were included in the 2010 Kenya Service Provision Assessment Survey (KSPA). The KSPA is a facility-based survey designed to provide information on the preparedness of health facilities that offer maternal, child, family planning, and reproductive health services, as well as services for specific infectious diseases. The components assessed are those commonly promoted in various programmes supported by the Government and development partners. The child health component of the survey was designed to assess the acceptability and perceptions of child caregivers about the new malaria vaccine, among other questions that provided countrywide information on child health during the time of the KSPA.

The sample of facilities included in the survey was selected from a *Master Facility List* (MFL) of 6,192 functioning health facilities in Kenya at the time of the survey. The list, obtained from the Division of Health Information Systems, Department of Standards and Regulatory Services, included hospitals, health centres, maternity and nursing homes, clinics, and stand-alone VCT facilities, run by the different managing authorities, including the Government of Kenya (GoK), non-governmental organizations (NGOs), private for-profit organizations, and faith-based organizations (FBOs). A sampling frame was used to randomly select a representative sample size of 703 health facilities from different levels, from the then eight provinces in Kenya. The sample was carefully designed to allow for key indicators to be presented at national and provincial levels, by type of facility, and by the different managing authorities. The sample covered approximately 11% of all facilities in the country, and included all three national referral hospitals and the eight provincial hospitals of Kenya. Data were weighted during the analysis to compensate for over- or under sampling of various types of facilities, in order to normalize the findings and present data from these facilities, as they exist in the country.

In the selected health facilities, caregivers of sick children were asked to participate in an Exit Interview as they left the facility. The Exit Interview included questions on the caregiver’s understanding and perception of the service delivery, and ascertained the caregiver’s attitude and perception about the new malaria vaccine.

### Data collection and analysis

A standard questionnaire was used for the interview. Interviewers sat in on consultations for 2,016 sick children and observed interactions between caregivers of the sick children and providers to assess whether the process followed in service delivery meets standards for acceptable content and quality. Only children younger than five years of age who presented with an illness (rather than an injury or a skin or eye infection exclusively) were selected for observation. Before leaving the facility, caregivers of observed sick children were interviewed about the perceived quality of the provider’s service, socio-demographic characteristics, and their attitude toward and perception of a new malaria vaccine. Demographic data for children whose caregivers were interviewed was also recorded, including the results of malaria tests done by either microscopy of a blood smear or by use of a rapid test kit. They were given the following information about the vaccine prior to answering the questionnaire:

• The vaccine would reduce the chances of getting severe malaria (e.g., malaria with convulsions) in a vaccinated child;

• The vaccine causes discomfort similar to other childhood vaccines;

• The vaccine may be given at the same health facility and at the same time as other childhood vaccines. The vaccine may require four to five jabs (shots) to receive full benefit;

• Because malaria can occur several times in a child, and because of how this vaccine is, it may not offer full protection against all episodes, that is, a child who is vaccinated with this vaccine could still get malaria;

• The vaccine does not change the need to prevent malaria through other ways. Even if the child is vaccinated, his/her family will need to continue malaria prevention practices (e.g., sleeping under an insecticide-treated bed net, taking prevention tablets during pregnancy, having the houses sprayed with insecticide, etc.)

The caregivers were then asked if they would support vaccination of a young child in the community with the future malaria vaccine. Those who had a child or children below five years were asked if they would have their own child vaccinated, and those with no children below five years were asked whether they would support the vaccination if they had a child below five years. All respondents were asked to give open-ended reasons why they would accept or not accept the malaria vaccine.

After completing data collection in each facility, there were different levels of quality assurance of data. Interviewers reviewed the questionnaires before handing them over to the team leader, who reviewed them a second time. A database developed using CSPro software was used for data entry. All questionnaires were entered twice to ensure accuracy. STATA v.11 was used for data analysis. Multinomial regression was applied to analyse the determinants of accepting a child to be immunized with the malaria vaccine.

### Ethical considerations

Informed consent was obtained from facility management, and from all interviewed participants. This study is a continuation of the qualitative study on community perceptions of a malaria vaccine that was approved by the African Medical Research Foundation (AMREF) Ethics and Scientific Review Committee. ICF Macro addressed all ethical issues and released anonymized data set from the Kenya Service Provision Assessment (KSPA).

## Results

### Socio demographic characteristics of caregivers

During consultations with sick children, a total of 2,003 caregivers completed the exit interviews between January and May 2010. Of the caregivers interviewed, 93.5% were female and 6.5% were male (Table 1). Most of the caregivers (77%) were in the prime reproductive age group of 20-34 years. Majority of the caregivers interviewed were related to the child; mothers made up over 90% of the caregivers, while fathers represented 6% and grandparents approximately 2%. Most caregivers interviewed were from the Western Province (17.4%) and the fewest were from Nairobi Province (7.0%). Most respondents (97%) took their children to health facilities at the district and lower levels, which primarily included dispensaries, followed by district hospitals and health centres. Overall, 72% of the caregivers were interviewed in government health facilities or those operated by local authorities (Table 1).

**Table 1:** Sociodemographic characteristics of caregivers interviewed and their distribution by region and facility type

**Characteristics of respondents**	**Frequency (n)**	**Per cent**
**Sex (n = 1,997)**		
Female	1,868	93.5
Male	129	6.5
**Age (n = 1,853)**		
<20	152	8.2
20-34	1,420	76.6
35-49	251	13.6
50+	30	1.6
**Relationship to child (n = 1,868)**		
Mother	1,686	90.3
Father	110	5.9
Other	72	3.8
**Region (n = 2,003)**		
Nairobi	141	7.04
Central	254	12.68
Coast	230	11.48
Eastern	285	14.23
North Eastern	196	9.79
Nyanza	291	14.53
Rift Valley	258	12.88
Western	348	17.37
**Facility type attended (n = 2,003)**		
National referral hospital	10	0.5
Provincial hospital	50	2.5
District hospital	365	18.2
Sub district hospital	309	15.4
Other hospital	286	14.3
Health centre	357	17.8
Clinic	119	5.9
Dispensary	443	22.1
Maternity	64	3.2

### Characteristics of children

Of the 2,016 sick children whose caregivers were interviewed, 53% were male and 47% were female (Table 2). One third of the children were infants less than 12 months old and approximately another third was between one and two years old.

**Table 2:** Characteristics of sick children

**Characteristic**	**Frequency**	**Percentage**
**Sex**		
Female	1068	53
Male	948	47
**Age (years)**		
<1	628	33.7
1-2	558	30
2-3	321	17.2
3-4	216	11.6
4-5	140	7.5
**Total**	**1,863**	**100**
**Malaria diagnosis (clinical)**		
Yes	752	38.1
No	1,221	61.9
**Total**	**1,973**	**100.0**
**Malaria test (blood smear or rapid test)**		
Positive	317	16.1
Negative	1,653	83.9
**Total**	**1,970**	**100.0**

Out of 1,973 children with fever, 752 (38.1%) were clinically diagnosed with malaria. However, of the 1,970 that underwent malaria testing using either microscopic blood smear examination or a rapid test kit, 317 (16%) of children tested positive (Table 2). The proportion of children who tested positive for malaria, in each province was as follows: 60% of cases in the Western Province, 48% in Nyanza, 42% in Eastern Province, 39% in the Coast region, 34% in the Rift Valley, and 33% in the North Eastern Province. The proportion in Nairobi and Central provinces were 13.5% and 13.7%, respectively.

### Caregiver responses to the potential malaria vaccination

Overall, about 88% of caregivers answered that they would support the vaccine for both a young child in their community and for their own child, while approximately 7% of respondents did not know whether they would support the vaccine and 5% would not support it (Table 3). Analysis of the distribution by province reveals that approval is highest in Nyanza (98.9%), Coast (98.7%), Eastern (97.8%), Central (96.7%), Western (95.4%), Rift Valley (91%) and Nairobi (87%). North Eastern Province had the lowest approval with 23% of caregivers stating that they would support child immunization with the possible malaria vaccine. Results were comparable for responses to vaccinating a child within the community and one’s own child (Table 3).

**Table 3:** Responses by caregivers to questions on the malaria vaccine

	**Support for young child in community to be vaccinated with the malaria vaccine**		**Support own child to be vaccinated with the malaria vaccine**	
	**Number (n)**	**Per cent**	**Number (n)**	**Per cent**
Yes	1,699	88.7	1,645	88.0
No	88	4.6	86	4.6
Don’t know	128	6.7	139	7.4
Total	1,915	100	1,870	100

Caregivers were asked to explain why they would accept or not accept a child to be immunized with the possible malaria vaccine. Over 92% of caregivers cited reduced mortality and morbidity as a reason they would accept their child, as well as a child in the community to be vaccinated. Furthermore, about 49% of caregivers who stated that they would not accept the vaccine cited the combination of possible side effects and incomplete protection provided by vaccines as reasons.

### Educational factors and acceptance of the vaccine

About 86% of caregivers interviewed had attended school in the past. Out of these, 94% would accept a child to be vaccinated in their community, while 3% would not and 3% did not know. Similar results were observed when caregivers were asked if they would accept their own child to be vaccinated (Table 4). Only 56% of those who had never attended school supported the vaccine in the community, and 55% for their own child.

**Table 4:** Caregivers’ educational factors and response to acceptance of the malaria vaccine

**Factors**	**Vaccination in community**				**Vaccination of own child**			
	**Yes**	**No**	**Don’t know**	**Total**	**Yes**	**No**	**Don’t know**	**Total**
	**% (n)**				**% (n)**			
**Ever attended school**								
Yes (n = 1,603, 85.9%)	94 (1,508)	3.4 (55)	2.6 (41)	100 (1,604)	93.3 (1,496)	4.2 (67)	2.5 (40)	100 (1,603)
No (n = 263, 14.1%)	55.9 (147)	12.6 (33)	31.6 (83)	100 (263)	55.1 (145)	7.2 (19)	37.6 (99)	100 (263)
Total (n = 1,866, 100%)	88.6 (1,655)	4.7 (88)	6.6 (124)	100 (1,867)	87.9 (1,641)	4.6 (86)	7.5 (139)	100 (1,866)
**Highest level of education**								
Primary (n = 959, 59.9%)	95.3 (915)	1.9 (18)	2.8 (27)	100 (960)	94.3 (904)	2.7 (26)	3.0 (29)	100 (959)
Secondary + (n = 641, 40.06%)	92 (590)	5.8 (37)	2.2 (14)	100 (641)	91.9 (589)	6.4 (41)	1.7 (11)	100 (641)
Total (n = 1,600, 100%)	94 (1,505)	2.6 (55)	2.6 (41)	100 (1,601)	93.3 (1,493)	4.2 (67)	2.5 (40)	100 (1,600)
**Literacy**								
Read and write (n = 769, 64%)	96 (738)	1.8 (14)	2.2 (17)	100 (769)	95.1 (731)	2.6 (20)	2.3 (18)	100 (769)
Read only (n = 49, 4.08%)	73.5 (36)	6.1 (3)	20.4 (10)	100 (49)	71.4 (35)	8.2 (4)	20.4 (10)	100 (49)
None (n = 384, 32%)	69.9 (268)	8.6 (33)	21.6 (83)	100 (384)	69.1 (265)	5 (19)	26 (100)	100 (384)
Total (n = 1,202, 100%)	86.7 (1,042)	4.2 (50)	9.2 (110)	100 (1,202)	85.8 (1,031)	3.6 (43)	10.7 (128)	100 (1,202)
**Paid fees for visit**								
Yes (n = 953, 51%)	91.2 (869)	4.7 (45)	4.1 (39)	100 (953)	90.8 (865)	4.9 (47)	4.3 (41)	100 (953)
No (n = 915, 49%)	86 (788)	4.7 (43)	9.3 (85)	100 (916)	85 (778)	4.3 (39)	10.7 (98)	100 (915)
Total (n = 1,868, 100%)	88.7 (1,657)	88	6.6 (124)	100 (1,869)	88 (1,643)	4.6 (86)	7.4 (139)	100 (1,868)

Of the caregivers who had attended school, 60% indicated that the highest level of education they completed was primary school. Out of these participants, 95% stated their support for the possible vaccine at the community level, and 94% supported its use for their own child. About 39% of caregivers had completed secondary school education or higher, and of this group, 92% approved of the vaccine in the community and a similar proportion approved for their own child.

No literacy was reported by 32% of caregivers. For those without literacy, only 70% would accept the malaria vaccine for the community and 69% for their own child. About 64% of caregivers were able to read and write. Out of these, 96% would accept a child in their community to be vaccinated with the malaria vaccine and 95% would accept their own child to be vaccinated. Four per cent of caregivers indicated that they could read but could not write, and 73.5 and 71.4% of these reported they would accept the malaria vaccine for the community and for their own child, respectively.

## Acceptance of the vaccine and factors related to the health facility attended

### Proximity to the health facility

About 78% of the caregivers who answered the question about vaccination of children in the community attended the health facility closest to their home. Among these, 87% would accept the malaria vaccine for the community and 91% for own child. Of those who did not attend the nearest facility, 94% accepted vaccine for the community and 85% for own child (Table 5). Out of those who did not go to the nearest health facility, 21% did so because the facility had a bad reputation or was more expensive, while 15% did so due to a lack of required medicines.

**Table 5:** Satisfaction of caregivers with the attended health facility and their response to acceptance of the malaria vaccine

**Factors**	**Vaccination in community**				**Vaccination of own child**			
	**Yes**	**No**	**Don’t know**	**Total % (n)**	**Yes**	**No**	**Don’t know**	**Total**
**Facility nearest home**								
Yes (n = 953, 51%)	87.2 (1,265)	4.9 (71)	7.9 (114)	100 (1,450)	90.8 (865)	4.9 (47)	41 (4.3)	100 (953)
No (n = 915, 49%)	93.5 (391)	4.1 (17)	2.4 (10)	100 (418)	85 (778)	4.3 (39)	10.7 (98)	100 (915)
Total (n = 1,868, 100%)	88.7 (1,656)	4.7 (88)	6.6 (124)	100 (1,868)	88 (1,643)	4.6 (86)	7.4 (139)	100 (1,868)
**Would recommend facility**								
Yes (n = 1,694, 91.9%)	90.9 (1,540)	4.1 (69)	5.1 (86)	100 (1,695)	90.3 (1,529)	4.3 (72)	5.5 (93)	100 (1,694)
No (n = 108, 5.9%)	77.8 (84)	7.4 (8)	14.8 (16)	100 (108)	75 (81)	9.3 (10)	15.7 (17)	100 (108)
Don't know (n = 41, 2.22%)	31.7 (13)	26.8 (11)	41.5 (17)	100 (41)	31.7 (13)	9.8 (4)	58.5 (24)	100 (41)
Total (n = 1,843, 100)	88.7 (1,637)	4.8 (88)	6.5 (119)	100 (1,844)	88.1 (1,623)	4.7 (86)	7.3 (134)	100 (1,843)
Total	1,651	88	121	1,860	1,637	86	136	1,859

### Recommendation of the health facility

Ninety-two per cent of caregivers would recommend the health facility that they attended to a friend. Out of these, 91% would accept malaria vaccination in their community and a similar percentage would accept vaccination of their own child. In comparison, only 78 and 75% of those who would not recommend the health facility would support the vaccine for community and own child, respectively (Table 5).

### Fee paid for service

About 51% of the caregivers paid fees for services received during their visit to the health facility. Of these, 91% would accept a child to be vaccinated with the malaria vaccine in their community or for their own children, compared to 86 and 85% acceptance for community or own child respectively, amongst those who did not pay fees for the services.

### Factors associated with acceptance of the malaria vaccine

A multivariate analysis to determine factors associated with a caregiver accepting a future malaria vaccine demonstrated that region of residence had a large influence on whether caregivers would accept a future malaria vaccine or not (Table 6). Residence in Central, Coast, Eastern, Nyanza, Rift Valley, and Western Provinces significantly increased the chances of a caregiver accepting a child to be vaccinated with the malaria vaccine in one’s community, as well as own child. On the other hand, a significantly reduced likelihood of accepting the vaccine for a child was found for residents of North Eastern Province.

**Table 6:** Regression of factors related to caregivers accepting vaccination of their own child

	**Relative risk ratio (RRR) of accepting own child to be vaccinated with possible malaria vaccine/Not accepting**		**RRR of not knowing (DK) whether to accept or not/Not accepting**	
**Factor**	**RRR**	**(95% CI)**	**RRR**	**(95% CI)**
**Region**				
Nairobi^R^	1.0		1.0	
Central	4.0**	(1.7, 11.7)	3.7**	(1.5, 9.3)
Coast	13.1***	(3.5, 48.9)	11.6***	(3.2, 42.5)
Eastern	8.7***	(3.0, 25.3)	5.7***	(2.2, 14.7)
North Eastern	0.3*	(0.1, 0.8)	0.4	(0.1, 1.1)
Nyanza	12.0***	(3.3, 43.3)	9.0***	(2.9, 28.3)
Rift Valley	3.2**	(1.4, 7.7)	2.4*	(1.1, 5.2)
Western	3.7**	(1.6, 8.3)	2.7*	(1.3, 5.6)
**Age (years)**				
<1	1.0			
1 - 2	1.3	(0.7, 2.4)	1.1	(0.6, 1.9)
2 - 3	1.4	(0.7, 2.9)	1.2	(0.6, 2.4)
3 - 4	1.1	(0.5, 2.4)	1.0	(0.5, 2.1)
4 - 5	1.2	(0.4, 3.1)	0.8	(0.3, 1.9)
>5	0.5	(0.0, 5.2)	0.5	(0.0, 4.7)
**Paid fees**				
Yes^R^	1.0		1.0	
No	1.3	(0.8, 2.2)	1.3	(0.8, 2.0)
**Nearest H/F**				
Yes^R^	1.0		1.0	
No	1.1	(0.6, 2.0)	0.8	(0.5, 1.4)
**Opinion on services**				
Satisfied^R^	1.0		1.0	
Somewhat satisfied	0.5**	(0.3, 0.8)	0.5**	(0.3, 0.9)
Not satisfied	0.6	(0.2, 1.9)	0.5	(0.2, 1.2)
**Relationship to child**				
Mother^R^	1.0		1.0	
Father	2.0	(0.7, 5.8)	1.4	(0.5, 3.6)
Other	3.3	(0.8, 13.2)	2.6	(0.5, 12.9)
**Age of caregiver**				
<20^R^	1.0		1.0	
20-34	0.8	(0.3, 2.0)	1.1	(0.5, 2.7)
35-49	0.3*	(0.1, 0.9)	0.5	(0.2, 1.4)
50+	0.1*	(0.0, 0.7)	0.4	(0.1, 3.0)
**Ever attended school**				
Yes^R^	1.0		1.0	
No	0.4*	(0.2, 0.9)	0.6	(0.3, 1.4)

Note: ***: p < 0.001; **: p < 0.01; *: p < 0.05.

Omitted categories (R in superscript) are as follows: Region: Nairobi; Age of child: 0-11 months; Paid fees: Yes; Nearest health facility: Yes; Opinion on services: Satisfied; Relationship to child: Mother; Age of caregiver: <20; Attended school: Yes.

Caregivers above age 35, particularly those above age 50, and those who never attended school were also significantly less likely to accept the vaccine for their own child. Being somewhat satisfied with the services at the health facility was associated with a significantly reduced likelihood of accepting the vaccine for both a child in one’s community and for their own child, compared to caregivers who reported being satisfied with health services (Table 6). Age of child, proximity of attended health facility, caregiver relationship to child and other socio-demographic factors were not associated with acceptance of a future malaria vaccine.

## Discussion

National decisions on the use of a new intervention require strong supportive data to best facilitate its timely and systematic uptake. It is critical to gain an understanding of the socio-cultural environment prior to introduction of a possible malaria vaccine, as in the past it has taken up to two decades for some vaccines to be available to communities in developing countries [7,18,19]. Upon investigating the acceptability and attitudes of child caregivers toward a malaria vaccine in all eight regions of Kenya, it was found that the majority of caregivers would support the introduction of a malaria vaccine. Previous studies in Kenya, Tanzania, Mozambique, and Ghana similarly demonstrated a widespread support for a possible new malaria vaccine [11-13]. Reduced mortality and morbidity were the main reasons stated for acceptance of the vaccine, whereas many would not accept the vaccine due to a combination of possible side effects and incomplete vaccine protection. This is congruent with previous findings that the more severe and visible the disease, and the more safe and effective the vaccine is perceived to be, the greater the acceptance and uptake of the new vaccine [7,19]. A study of vaccination of children with seasonal influenza vaccination in Kenya also demonstrated that parents being too busy or their child being away during the vaccination period were reasons for having declined the vaccine [19].

### Socio-demographic factors underlying acceptance of the vaccine

The majority (77%) of the caregivers were in their early and mid-reproductive years (between 20 and 34 years of age). The results of this study showed that beyond 35 years of age, the chance of a caregiver accepting their child to be vaccinated with the possible malaria vaccine decreased progressively with age (RRR 0.3 for age group 35-49 years, compared to 0.1 for age 50 years and above). This finding is in agreement with other studies, which have shown that children of caregivers aged above 40 years were more likely to be lag behind in the vaccination schedule [10,20-22].

Over 90% of the caregivers were mothers, confirming the prominent role mothers play in taking care of sick children. This is in line with the observation of the WHO that women are the main conduits for health knowledge, the main providers of health care [23]. Even when both parents are working, the responsibility of care for sick children usually falls on the mother [23,24]. This underscores the need to empower women to make timely healthcare decisions and enable them to execute these.

### Regional context for uptake of a possible malaria vaccine

Findings from this study show that regional disparities, caregiver opinion on the service, age of the caregiver, and school attendance play significant roles in having caregivers accept immunization of children in Kenya. For example, being a resident of North Eastern Province is significantly associated with a reduced risk (0.3) of accepting vaccination. These findings are consistent with those of the Kenya Demographic and Health Survey (KDHS), which show that only 48% of children aged between 12 and 23 months in North Eastern Province received all basic vaccinations [14].

The low likelihood of accepting the vaccine in this Province may be a result of many factors underlying poor vaccination support and coverage in the socio-economically disadvantaged North Eastern Province, including poverty, lack of knowledge, poor health awareness, poor access to healthcare, lack of infrastructure and religious considerations [25,26]. North Eastern Province, located in the drought prone arid lands of northern Kenya is one of the poorest regions in the country where majority live below the poverty line, has some of the worst health indicators in the country, vaccination coverage is low and school enrolment is low [14,27].

A WHO report [10] identified various immunization system, family characteristics and parental attitude and knowledge related barriers to vaccine uptake in Kenya. Immunization system related reasons/factors linked to un-vaccinated or under-vaccinated children included: cost of access to immunizations, inconvenient clinic schedules, increased distance from vaccination clinic, other parental duties, missed opportunities due to vaccines or supplies stock outs, lost vaccination card, or child was sick or under weight. Older mothers; low level of education of mother; divorced mothers; children in large families and parents with large families were family factors associated with low vaccine uptake. On the other hand, the parental attitude and knowledge related factors associated with low vaccine uptake included very low (or very high) knowledge level regarding immunizations; negligence and/or ignorance among caregivers regarding children's vaccinations; caregivers too ‘busy’ to take children to get vaccinations; parental beliefs; lack of motivation of parents and lack of knowledge of disease. Many of the factors identified here obtain in North Eastern Province and may contribute to the low levels of acceptance of a malaria vaccine. For instance, about 80% of girls in the province are not enrolled in school [27] and only 21% of women in the province are literate, compared to 65% of the men and the overall national average of 87% [14]. This low literacy levels among women, who are the primary caregivers of children, may play an important role in the low acceptance of a malaria vaccine in North Eastern Province.

In Karachi, Pakistan, Sheik *et al*. identified the most common reasons for non-vaccination as a lack of knowledge, as well as non-compliant spouse, security conditions, religious taboos, lack of trust in medical facilities, fear of side effects, accessibility problems, financial problems, and vaccinations being considered as ineffective [28]. Karachi and North Eastern Province in Kenya share religious similarities, as well as comparable regional challenges. There is a dire need for concerted efforts by the central and county governments to address the socio-economic disparities in the province, in order to improve acceptability and equitable access to health services, including vaccines.

The study found that being a resident of Coast, Nyanza, Western, Central and Eastern Provinces is significantly associated with a higher likelihood of accepting a child to be immunized. As expected, the highest number of malaria-positive tests was also found in the malaria-endemic regions of Western, Nyanza and Coast Provinces, including the seasonal malaria transmission Eastern Province. High approval rates in these regions are likely related to residents’ desire to support interventions, which promise to reduce the malaria burden in their community. Yet, most caregivers in both malaria-endemic and non-endemic areas would approve vaccination of children with a malaria vaccine with the exception of the North Eastern Province. In the case of the low-risk malaria regions of Central and Nairobi Provinces, this may be explained by greater health awareness and higher literacy levels in these regions. The fact that people often move into and out of malaria-endemic zones may also provide a reason why widespread approval of a possible vaccine cuts across the entire country.

However, the low levels of acceptance witnessed in North Eastern Province may be due to the general low uptake of vaccines in the province, related to the factors mentioned above. On the other hand, it may be linked to the fact that the area lies in the seasonal malaria transmission zone where the periods of malaria transmission during the rainfall seasons are short. The province generally has low population densities and has traditionally not been targeted for scaling of malaria preventive interventions; exhibit highly focal transmission close to water features [29]. Because of the presumed low malaria risk, few empirical studies of malaria have been undertaken in these areas and the malaria situation among these poor, pastoralist communities remains ill-defined. The Kenya Malaria Indicator Survey of 2010 [30] found that the level of knowledge of malaria disease, perception of severity of fever, health seeking behaviour for fever, perceived affordability and availability of first line anti-malarials, and perceived efficacy of anti-malarials in treating fever were related to malaria endemicity. Women of reproductive age from the semi-arid seasonal malaria transmission zone that includes North Eastern Province were less likely to know the first line treatment for malaria; were likely to perceive fever as a little serious or not serious at all; less likely to seek prompt anti-malarial treatment; fewer agreed that fever treatment was available, and that malaria medicines were affordable and effective, compared with mothers in higher risk malaria endemicity zones. In addition, distance to services has been identified as a reason for low vaccine uptake by caregivers living in rural and/or remote communities, often in locations without a health facility [10]. The low knowledge of malaria disease and low perception of malaria risk by caregivers, coupled with poor access to health facilities in North Eastern Province may be linked to the low acceptance of a malaria vaccine. A lot needs to be done to improve health awareness and access to health services in the North Eastern Province.

### Influence of education on caregiver perceptions

Regression results showed that not having attended school significantly reduced the likelihood of a caregiver accepting their child to be immunized with a malaria vaccine than those who were not schooled. Similarly, a higher rate of acceptance was found among respondents who could read and write, compared to those without literacy. This finding is consistent with previous studies that showed that children whose caregivers were educated were more likely to complete their immunization schedule on time [10,31]. This could be attributed to various factors, including greater health awareness with better utilization of health facilities, greater involvement in decision making regarding child health, ability to accept new ideas, and greater confidence when dealing with health professionals [32]. A recent study from Australia also found that parental confidence in vaccine safety was significantly associated with a higher level of education [33].

A randomized controlled trial in Karachi, Pakistan demonstrated that home-based education for mothers on the importance of vaccines significantly improved infant immunization rates in low-income and low-literacy populations [34]. Another trial concluded that provision of an improved immunization card, centre-based maternal education, or both interventions together improved childhood immunization adherence [35]. Such immunization promotion programmes should be considered in the North Eastern Province and other low-literacy areas.

### Impact of satisfaction with health care services on child immunization

Being somewhat satisfied with the services offered in a health facility was significantly related to a reduced likelihood (RRR 0.3, p < 0.01) of accepting one’s own child to be vaccinated with a future malaria vaccine. This finding is in tandem with results from a qualitative study that was conducted in Coast and Busia regions, where access to vaccination services and attitudes of service providers were mentioned as barriers to immunization [13].

### Limitations

This was not a household survey and the caregivers present on the day of the survey at the selected facilities might not be representative of the caregivers in the community who normally seek immunization services. Furthermore, being a hospital-based survey, perceptions of caregivers on vaccines could be influenced by their experience in using health services, the quality of their interaction with health providers, as well as the services received. Additionally, caregivers were asked why they would decline or accept a malaria vaccine, yet the responses were too general, e.g., “reduces mortality”. In future studies, this question should be open-ended to allow for more thorough and informative responses.

During the data collection, the country was stratified along provincial administrative units but not according to malaria endemicity zoning. The study cannot therefore conclusively relate acceptance of a malaria vaccine to malaria endemicity zones. Future studies should explore the correlation between acceptance of a malaria vaccine and malaria endemicity zones.

It should be noted that this study assessed caregiver perceptions of a malaria vaccine assuming that it would be sufficiently efficacious to “reduce the chances of getting severe malaria in a vaccinated child”. Other studies have highlighted widespread preference of a malaria vaccine over drugs, provided that the vaccine is efficacious. At the time this study was conducted, efficacy results from clinical trials were limited and in the meantime, relatively lower efficacy has been found for RTS,S compared to other childhood vaccines [5]. It will therefore be valuable to assess caregiver perceptions of the vaccine with final efficacy results and information from the trials as they conclude. Despite these limitations, this study demonstrates that introduction of a vaccine for malaria has overwhelming support from caregivers of sick children who visited health facilities.

## Conclusion

The results of the study show high endorsement and expectations of malaria vaccination in all regions of the country. The exception is North Eastern Province, where the possibility of accepting one’s own child to be vaccinated with this future vaccine is low. The overwhelming support calls for the need to carefully manage the expectations of end users as the vaccine is released in the future. The results also point to the need to target specific segments of child caregivers with relevant messages. Some of these segments include residents of regions where acceptance is low, service providers in health facilities, older caregivers, and those who have not received an education.

## Abbreviations

WHO: World Health Organization; MVI: Malaria Vaccine Initiative; KSPA: Kenya Service Provision Assessment Survey; GoK: Government of Kenya; NGOs: Non-governmental organizations; FBOs: Faith-based organizations; MFL: Master Facility List; AMREF: African Medical Research Foundation; RCT: Randomized Controlled Trial; KDHS: Kenya Demographic and Health Survey.

## Competing interests

The authors declare that they have no competing interests.

## Authors’ contributions

DIO is the principal investigator on the study, and acquired the data, completed data analysis and provided edits to the manuscript. JDJ and MIM wrote and edited the manuscript. ST contributed to writing and inputs for the original research report from which this paper was drafted. All authors read and approved the final manuscript.
